# Reasons to be fearful? Rising proportions of positive faecal worm egg counts among UK horses (2007–2023)

**DOI:** 10.1111/evj.14478

**Published:** 2025-01-22

**Authors:** Fleur Whitlock, Jan van Dijk, Jane E. Hodgkinson, John Duncan Grewar, J. Richard Newton

**Affiliations:** ^1^ Equine Infectious Disease Surveillance (EIDS), Department of Veterinary Medicine University of Cambridge Cambridge UK; ^2^ Animal Health Vision International Taunton UK; ^3^ Institute of infection, Veterinary and Ecological Sciences University of Liverpool Neston UK; ^4^ jDATA (Pty) Ltd Sandbaai South Africa

**Keywords:** anthelmintic resistance, horse, parasitology, surveillance

## Abstract

**Background:**

Anthelmintic resistance (AR) threatens effective equine parasite control. Quarterly data summaries from faecal worm egg count testing (FWECT) performed by UK laboratories have appeared in Equine Quarterly Disease Surveillance Reports (EQDSR) since 2007, but have not previously been assessed.

**Objectives:**

To assess strongyle FWECT methods and thresholds used by UK laboratories. To investigate factors associated with quarterly laboratory FWECT positivity rates between 2007 and 2023.

**Study design:**

Laboratory surveys and analysis of laboratory summary data.

**Methods:**

Laboratories were surveyed in Q3 2018 and again in Q4 2023. Proportions of FWECTs reported positive (PTP) each quarter by individual laboratories between 2007 and 2023 were analysed using multiple mixed‐effects linear regression, evaluating laboratory‐level random‐effects and fixed‐effects variables for ordered categories of FWECT‐thresholds, year‐quarters and consecutive year groups.

**Results:**

Ten laboratories responded in 2018 and 13 laboratories in 2023. Samples were commonly reported positive at >0 to <100 eggs per gram (epg) and ≥200 epg. Regression modelling of 1190 EQDSR submissions confirmed significantly decreased PTP for thresholds ≥100 to <300 epg (level‐2: −12.0%, *p* = 0.03), ≥300 epg (level‐3: −18.0%, *p* = 0.03) and when thresholds were not specified (level‐4: −12.2%, *p* = 0.0), relative to level‐1 baseline (>0 to <100 epg). No significant seasonal variation in PTP between year‐quarters was evident. Overall, controlling for between‐laboratory variation and FWECT thresholds, there remained evidence for a significant gradient in increasing PTP over the study period relative to baseline (2007–2009). There were increases in PTP of +6.9% in 2010–2011 (*p* < 0.001), +10.1% in 2012–2013 (*p* < 0.001), +14.1% in 2014–2015 (*p* < 0.001), +16.0% in 2016–2017 (*p* < 0.001), +15.6% in 2018–2019 (*p* < 0.001), +17.1% in 2020–2021 (*p* < 0.001) and +18.9% in 2022–2023 (*p* < 0.001).

**Main limitations:**

Survey responses were limited and most laboratories' FWECT thresholds were not known.

**Conclusions:**

Controlling for laboratories and FWECT thresholds there was strong residual evidence from FWECT summary data for increasing egg counts in UK horses between 2007 and 2023.

## 1 INTRODUCTION

Anthelmintic resistance (AR) is an ever‐present global issue that requires addressing to ensure the effectiveness of worm control now and in the future.^1^, ^2^, ^3^, ^4^, ^5^ The recently developed United Kingdom industry‐wide initiative, CANTER (Controlling ANTiparasitic resistance in Equines Responsibly) is working to generate a coordinated approach for the equestrian community to combat AR.^6^ Surveillance analysis, to inform best practices and responses to any strategies that may be taken by the industry in an attempt to address the issue of AR, is essential. The Equine Quarterly Disease Surveillance Report (EQDSR) is produced by Equine Infectious Disease Surveillance (EIDS) at the University of Cambridge, on behalf of the Department of Agriculture, Food and Rural Affairs (Defra), Animal and Plant Health Agency (APHA) and the British Equine Veterinary Association (BEVA).^7^ The EQDSR compiles and shares information from surveillance schemes covering different equine infectious diseases across the United Kingdom. One particular focus within the report is laboratory testing surveillance, which summarises data from ~30 UK‐based equine diagnostic laboratories. Faecal worm egg count test (FWECT) results are included within this section and provide a unique resource for national equine parasite surveillance.

FWECTs use faecal samples from individual horses in an attempt to evaluate their burden of gastrointestinal parasitic nematodes based on enumerating the density of parasite eggs present in those samples. They are considered reliable for estimating strongyle egg shedding due to consistent shedding levels in individual horses over time.^8^, ^9^ However, the relationship between strongyle egg counts and adult worm burdens is poorly understood, with no clear association observed and no statistical evaluations conducted.^8^, ^10^, ^11^ Also, FWECTs for determining ascarid infection remain unevaluated.^11^ In the United Kingdom, faecal samples can be submitted directly to numerous laboratories by equine keepers, via the postal system, as well as testing conducted under veterinary supervision, with testing costs usually not exceeding those of anthelmintic products. FWECT results help provide information on whether a horse is infected with a nematode and what level they are shedding eggs at, which is key to aiding decisions around identifying which horses are contaminating pastures and therefore warrant anthelmintic treatment.^12^ It is increasingly important to use FWECTs to guide worming strategies, due to the emergence of AR. The goal is to use anthelmintic drugs only when necessary, to minimise the selection pressure which allows resistant worms to multiply.^13^, ^14^ Although numerous methods for FWECTs exist, there is currently no standardisation and no regulation or accreditation for laboratories to subscribe to in the United Kingdom. There are several methods used for conducting FWECTs^15^ (Table 1), with the most well‐known being the McMaster method, with or without modifications,^16^, ^17^ as well as other methods, including the Ovatec (Zoetis UK Ltd)^18^ and the OvaCyte telenostic analyser.^19^

**TABLE 1:** Summary of different FWECT methods.

FWECT method	Description	Origin
McMaster	Mix faecal sample with water and flotation fluid (such as Sheather's sugar solution or saturated sodium chloride solution). Flotation fluid allows eggs to rise up to under the surface of a glass slide, so all eggs are in the same focus and debris is out of focus on the floor of the chamber.	Gordon and Whitlock^16^
Modified McMaster	Variety of modifications made to improve the McMaster method. Modified by the weight of faeces or the volume or type of flotation solution prepared.	Various^17^
Ovatec™	Device for quicker and easier worm egg counting.	Zoetis UK Ltd.^18^
OvaCyte telenostic analyser	Digital microscope linked to artificial intelligence for automated worm egg counting.	Telenostic; in collaboration with University College Dublin's Department of Parasitology and Department of Engineering, and the Irish Centre for High End Computing^19^

In light of the recent imperatives for and advancements in combatting AR, surveillance data summarising FWECT results such as those in the EQDSR, provide a potentially significant resource. In this article, we looked to determine the variation in testing methods used across the laboratories contributing to the EQDSR, including the thresholds used by a laboratory to report a sample as positive for surveillance and treatment purposes. Additionally, we evaluated the FWECT positivity rate over a 17‐year period to determine trends and provide a baseline for future comparisons, such as after issuing future advisories aimed at combatting AR. We looked to test the hypothesis that, after controlling for the adoption of different laboratory positivity thresholds and potential seasonal effects, there remains a significant temporal trend over the study period for increasing PTP.

## 2 MATERIALS AND METHODS

### 2.1 Survey of FWECT methods and thresholds used by laboratories submitting EQDSR data

Data on how FWECTs were conducted by different laboratories across the United Kingdom, and how their results were reported to the EQDSR and clients, were collected at two time points, separating answers for strongyles and ascarids. Initially, a request for surveillance reporting thresholds was communicated to all laboratories contributing to the EQDSR in Q3 2018. Later, a formal questionnaire containing more in‐depth questions around testing methodology and thresholds for surveillance contributions and treatment levels was sent to 17 laboratories across the United Kingdom that contributed FWECT data to the EQDSR between October and December 2023, with a reminder sent to non‐responders over this time (Questionnaire S1).

### 2.2 Summary FWECT surveillance data submitted by laboratories to EQDSR

Summary FWECT data were collated between 2007 and 2023, representing the total number of faecal samples tested by individual laboratories each quarter, along with the number of samples deemed positive (i.e., above each laboratory's positive threshold), as absolute counts of FWECTs were unavailable.

### 2.3 Data analysis

Laboratory questionnaire data were collected in Microsoft Word (version 2019) and subsequently inputted and stored in Microsoft Excel (version 2019). EQDSR FWECT data were inputted and stored in PostgreSQL (PostgreSQL Global Development Group—www.postgresql.org) database. Exploratory data analysis was performed in R using RStudio version 2024.04.0 with the use of additional packages including RPostgreSQL, dplyr and ggplot2 and Stata Statistical Software version 18.

Descriptive data were produced to summarise FWECT methods and FWECT thresholds used, with percentages used to describe and analyse data. The percentage of strongyle FWECTs reported positive (‘Percentage of Tests Positive’ shortened hereafter to PTP) for each laboratory's data submission for each quarter of each year was calculated (number of samples positive/number of samples tested). To investigate and ultimately control for the effect of the different eggs per gram (epg) positive threshold values used by laboratories to report a sample as positive for surveillance purposes to the EQDSR, the data for each laboratory were categorised according to the threshold values of each laboratory used (where this information was provided in the survey response). This resulted in four categories of threshold levels adopted by contributing laboratories that were used in subsequent analyses, comprising where individual laboratories threshold level was between ≥0 and <100 epg (level 1, referent baseline), between ≥100 and <300 epg (level 2), ≥300 epg (level 3) and NA being the group for laboratories for which the threshold was not known (level 4). Data were analysed using these subsets to determine the (i) total (*n*) tests performed, (ii) total (*n*) tests reported positive and (iii) overall PTP. FWECT PTP for each year for all positive cut‐off threshold groups combined and as individual groups were visualised in box and whisker plots. Normality of distribution of PTP was evaluated through visual assessment of a kernel density plot and a mixed‐effects linear regression analysis was used to model the PTP of samples at the individual laboratory level accounting for several factors, including year‐quarter (Q1: January–March, Q2: April–June, Q3: July–September and Q4: October–December), study period categorised in eight ordered categories (2007–2009, 2010–2011, 2012–2013, 2014–2015, 2016–2017, 2018–2019, 2020–2021 and 2022–2023), positive cut off threshold and laboratory identity, with individual laboratories (*n* = 36) modelled as a random effect. The fixed effects variables were set with their baseline categories as referent, that is, threshold level 1, Q1: January–March and the period 2007–2009. Laboratory quarterly summary returns to EQDSR where no tests were performed or positive samples and PTP were not determined were excluded, including a laboratory (H) that adopted a positive FWECT threshold of ≥300 epg but did not return the number of positive results and for which PTP could not be determined. Initially, all three variables were examined univariably (year‐quarter, positive cut‐off threshold level and laboratory identity). Subsequently, a forward step‐wise approach was used to build the multivariate model. Variables were retained in the final regression model when Wald chi‐square *p*‐values achieved statistical significance at *p* ≤ 0.05.

## 3 RESULTS

### 3.1 Survey of FWECT methods and thresholds used by laboratories submitting EQDSR data

There were 10 laboratories that responded in 2018 and 13 of 17 (76.5%) laboratories responded to the questionnaire shared in Q4 2023, with five of these responding on both occasions, giving 18 laboratories for which FWECT data were available. Of the 13 respondents in 2023, 12 (92%) laboratories indicated that they conducted FWECT using the McMaster method, with or without modification, two also offered other methods such as the OvaCyte Telenostic analyser and Ovatec and one laboratory only used the Ovatec method (Table 2).

**TABLE 2:** Information provided by 13 UK equine diagnostic laboratories surveyed in Q4 2023 on methods used when conducting FWECT.

Lab	FWECT method	Volume of faeces requested	Volume of faeces used in testing^a^	Multiplication factor applied to determine epg
A	Modified McMaster	Minimum 3 g, ideally 6 g	Unknown	×100
B	Modified McMaster and OCT	Minimum 10 g	3 g	×30
C	McMaster and Ovatec	Golf ball‐sized	Unknown	McMaster ×50 Ovatec ×25
D	McMaster	12 g from various places	3 g	×25
E	Modified McMaster	A handful/50 g from various places	Unknown	×100 or ×50
F	McMaster	>5 g	Unknown	×100
G	McMaster	>10 g	3 g	×100
H	McMaster	Large pinch	Unknown	×25
I	Modified McMaster	Unknown	Unknown	Unknown
J	Ovatec	2 g	Unknown	×10
K	McMaster	‘Golf ball sized’ from various places	Unknown	×50
L	Modified McMaster	5 large pinches from various places	Unknown	×50
M	Modified McMaster	Minimum 5 g	Use all of what is sent	×50 or ×25

Abbreviations: epg, eggs per gram; OCT, OvaCyte Telenostic analyser.

^a^ Not explicitly asked within the questionnaire but provided by some laboratories.

There was a wide variation in the volume of faeces that laboratories asked clients to submit, ranging from 2 g to a ‘golf ball‐sized’ sample. Requests for the faecal sample to be obtained from various areas across a dropping were specified by four laboratories (31%). Some laboratories responded to the survey with additional unrequested information in which they specified the volume of faeces they use in each FWECT including three laboratories that all used 3 g and one laboratory that processed all the samples submitted. With regards to the multiplication factor used on the microscope to estimate epg, this varied from ×10 to ×100, and was often reported to be dependent on the method of testing being used.

Overall, responses were provided by 18 laboratories for FWECT‐thresholds used to designate samples as strongyle egg positive for the purposes of reporting to the EQDSR (Table 3). Thresholds ranged between >0 and ≥400 epg, with ≥100 to <300 epg adopted by eight (44%) laboratories, 50 epg or less used by seven (39%) laboratories and ≥300 epg adopted by three (17%) laboratories. The five repeated laboratory responses in 2018 and 2023 were all concordant in the thresholds that they reported on the two occasions. Nine laboratories specified a threshold at which they would advise treatment for strongyles, with this level ranging from ≥200 to ≥500 epg, with ≥200 epg being most common and used by six laboratories. Of the four laboratories that did not specify a threshold for strongyle treatment, two were providing results to referring veterinary practices to interpret for themselves and two required a veterinary surgeon to determine if worming was required. Five laboratories specifically stated that a veterinary surgeon would advise the client on next steps, that is, if an anthelmintic treatment is required or any supplementary management changes.

**TABLE 3:** Information provided by 18 UK equine diagnostic laboratories on epg threshold levels and advisory comments used when conducting FWECT.

Lab	Epg for strongyle EQDSR positive	EQDSR threshold level group^a^ (1–4)	The epg for ascarid EQDSR positive	The epg level for advising strongyles treatment	The epg level for advising ascarid treatment	Comments used when reporting results	Most recent timepoint for data acquisition
A	50	1	50	500	500	Vet advises	Q4 2023
B	30	1	30	N/A	N/A	Results are sent to the referring practices	Q4 2023
C	200	2	200	200	200	Depends on results, horse age, health, management, time of year etc.	Q4 203
D	25	1	25	N/A	N/A	Results are sent to the referring practices	Q4 2023
E	>0	1	>0	Vet advises	Vet advises	Vet advises	Q4 2023
F	50	1	Not conducted	300	Not conducted	Vet advises	Q4 2023
G	100	2	100	200	200	None as no history provided	Q4 2023
H^b^	300	3	300	300	300	Use guidance on low, medium and high levels. Additional comments added are determined by the season	Q4 2023
I	200	2	Unknown	Vet advises	Vet advises	Vet advises	Q4 2023
J	200	2	200	200	200	Vet advises	Q4 2023
K	50	1	50	200	200	Vet advised. Recently introduced a form for owners to fill in to guide advice for worming. Use guidance on low, medium and high levels and have management discussions	Q4 2023
L	50	1	50	200	200	Use guidance on low, medium and high levels and have management discussions with a vet. Additional comment to advise testing for encysted red worm and tapeworm as this test does not detect the presence of these parasites	Q4 2023
M	200	2	50	200	50	Use guidance on low, medium and high levels and advise accordingly	Q4 2023
T	200	2	N/A	N/A	N/A	N/A	Q3 2018
V	300	3	N/A	N/A	N/A	N/A	Q3 2018
AA	100	2	N/A	N/A	N/A	N/A	Q3 2018
AC	400	3	N/A	N/A	N/A	N/A	Q3 2018
AF	200	2	N/A	N/A	N/A	N/A	Q3 2018

*Note*: Data collected at two time points (Q3 2018 and Q4 2023), with consistency in data confirmed when data was provided by five laboratories at both time points.

^a^ 1 = >0 to <100, 2 = ≥100 to ≤300 epg, 3 = ≥300 epg.

^b^ Noting that absence of data on numbers of tests positive and hence PTP, provided by this laboratory precluded inclusion of their data in EQDSR and subsequent analyses here (EQDSR, equine quarterly surveillance report).

Four laboratories indicated they followed guidance on low, medium, and high epg levels in their comments on test results, though no specific epg thresholds for these categories were provided. There were two laboratories that did not indicate that they conducted ascarid FWECT and of the 10 that did, nine used the same thresholds that they used for strongyles to report to the EQDSR and one laboratory used ≥50 epg for ascarids and ≥200 epg for strongyles.

### 3.2 Summary FWECT surveillance data submitted by laboratories to EQDSR

**FIGURE 1:**
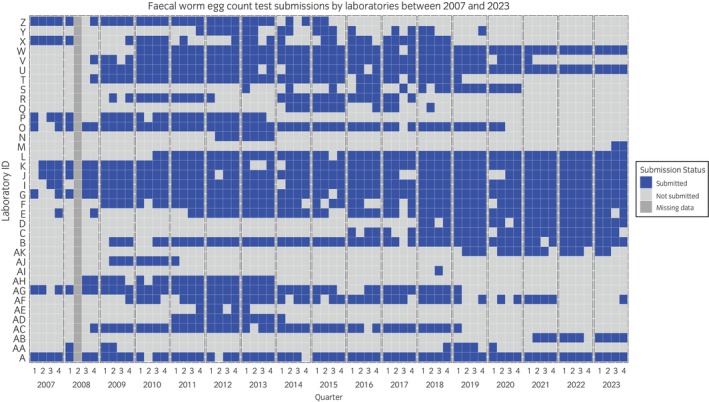
Data submissions to the EQDSR by 36 UK laboratories from 2007 to 2023 (no archived data available for Q2 2008 only). The heatmap illustrates the timeline and submission status of FWECT by each laboratory over the specified period.

**FIGURE 2:**
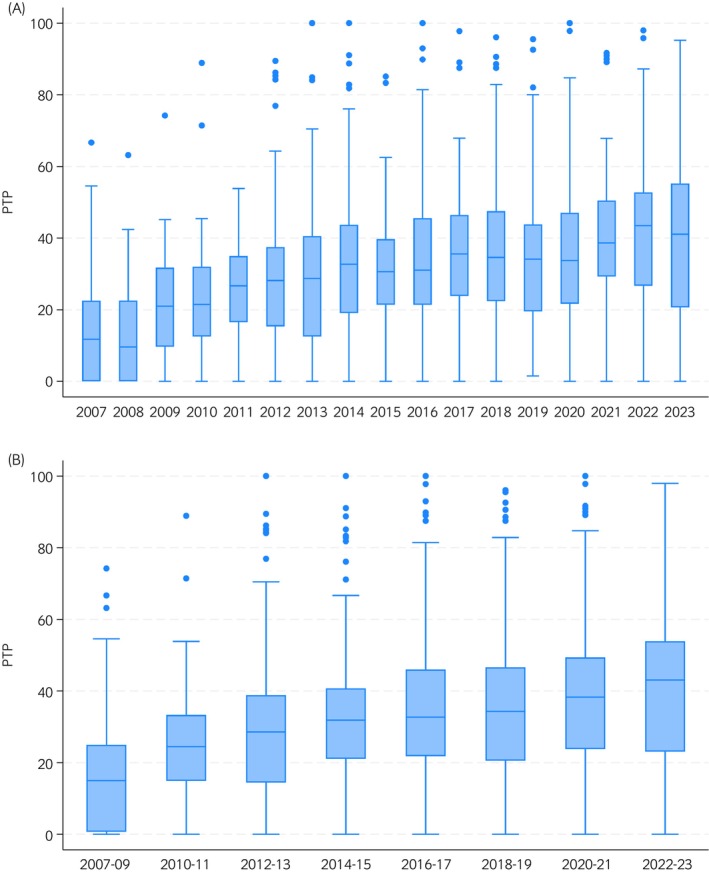
Box and whisker plots showing the distributions of percentages of strongyle FWECT reported positive (PTP) for (A) each year and (B) grouped years. Data were submitted quarterly by 36 UK equine diagnostic laboratories to the EQDSR between January 2007 and December 2023 (*n* = 1190).

Between 2007 and 2023 (with no archived data available for Q2 2008 only), 36 laboratories in the United Kingdom contributed FWECT number of samples tested and number positive data to the EQDSR at various points (Figure 1). Of these, 18 laboratories provided data for at least 30 quarters (7.5 years) and the 12 laboratories that also provided survey data on the FWECT methods and thresholds contributed a median of 54 quarters (13.5 years) of EQDSR data (IQR 42.7–63 quarters, range 2–64 quarters). All individual laboratories' quarterly summary FWECT results, regardless of the FWECT methods used, were included in analyses and distributions of PTP for each year and grouped years are depicted in the box and whisker plots in Figure 2.

**FIGURE 3:**
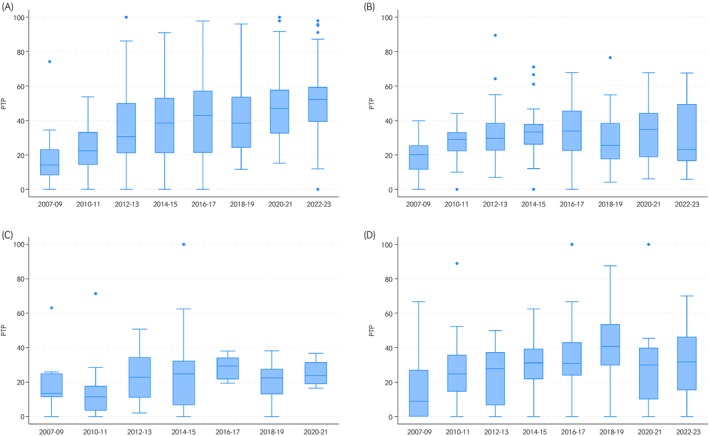
Box and whisker plots showing the distributions of percentages of strongyle FWECT reported positive (PTP) for grouped years, by laboratories' epg threshold groups. Data were submitted quarterly by 36 UK equine diagnostic laboratories to the EQDSR between January 2007 and December 2023. A = threshold level 1 (>0 to <100 epg; *n* = 358), B = threshold level 2 (≥100 to <300 epg; *n* = 306), C = threshold level 3 (≥300 epg; *n* = 88) and D = threshold level 4 (NA = for all laboratories for which the threshold was not determined; *n* = 438).

The median PTP for all submissions over the 17‐year period was 30% (IQR 17%–43%, range 0%–100%). The dataset was then grouped by categorising laboratories according to the FWECT threshold they employ to report data to the EQDSR (Table 4, Figure 3).

**TABLE 4:** Summary of testing frequencies, categorised by groups of the epg threshold used by laboratories to report a faecal worm egg count sample as positive to the EQDSR.

Threshold group^a^	Threshold level used (epg)	Number of laboratories	Total number of tests performed	Total number of tests reported positive	Overall percentage of tests positive (PTP) (%)
1	>0 to <100	7	68 602	30 218	44.0
2	≥100 to <300	8	195 243	52 423	26.9
3	≥300	2	15 532	3375	21.7
4 (NA)	Unknown	19	67 494	18 877	28.0
*Total*	—	*36*	*346 871*	*104 893*	*30.2*

^a^ NA = unknown epg threshold level, comprising three non‐responders to both information requests and 16 laboratories that are no longer active wrt data submission due to name change, merger or closure.

There were 1190 quarterly laboratory surveillance report submissions of PTP submitted by 36 individual laboratories that were included in the final mixed effects linear regression (MELR) model (Table 5). There was no evidence of a significant seasonal variation in PTP between year‐quarters and consequently these were not retained in the final MELR model. When interpreting the outputs for laboratory threshold level used to report to the EQDSR, compared with the baseline category of level 1 (>0 to <100 epg), all other categories had statistically significant negative coefficient estimates, consistent with reduced PTPs. There was an evident trend of reduction of −12% for ≥100 to <300 epg (level 2) and −18% for ≥300 epg (level 3) compared with baseline, consistent with a logical gradient of increasing threshold levels reducing PTP. Among the group of 19 laboratories where threshold levels were not known, there was also a significant reduction in PTP (−12%) compared with baseline. The year group categories (Figure 2, panel B) showed that PTPs increased statistically significantly along a clear gradient across the 17‐year period. Relative to baseline in 2007–2009 there were increases in PTP of +6.9% in 2010–2011 (*p* < 0.001), +10.1% in 2012–2013 (*p* < 0.001), +14.1% in 2014–2015 (*p* < 0.001), +16.0% in 2016–2017 (*p* < 0.001), +15.6% in 2018–2019 (*p* < 0.001), +17.1% in 2020–2021 (*p* < 0.001) and +19.0% in 2022–2023 (*p* < 0.001). A highly statistically significant (*p* < 0.001) random effect term confirmed an anticipated residual variation in PTP between different laboratories, that was not otherwise explained by the inclusion of year group and poorly defined threshold levels in the final model.

**TABLE 5:** Results of multivariable mixed effects linear regression analysis of percentage of FWECT with positive results (PTP), with laboratory identity (*n* = 36) included as a statistically significant (*p* < 0.0001) random effect variable (*n* = 1190).

Variable	Category	Coefficient	Coefficient standard error	95% confidence interval of regression coefficient	*p*‐value
Intercept		29.1	4.27	20.8 to 37.5	<0.001
Year group	2007–2009	Referent			
	2010–2011	+6.9	1.88	+3.2 to +10.6	<0.001
	2012–2013	+10.1	1.88	+6.4 to +13.8	<0.001
	2014–2015	+14.1	1.96	+10.2 to +17.9	<0.001
	2016–2017	+16.0	1.98	+12.1 to +19.8	<0.001
	2018–2019	+15.6	1.95	+11.7 to +19.4	<0.001
	2020–2021	+17.1	2.12	+12.9 to +21.2	<0.001
	2022–2023	+19.0	2.18	+14.7 to +23.2	<0.001
Threshold epg group	1: >0 to <100 epg	Referent			
	2: ≥100 to <300 epg	−12.0	5.62	−23.0 to −1.0	0.032
	3: ≥300 epg	−18.0	8.53	−34.8 to −1.3	0.034
	4: NA	−12.2	4.79	−21.6 to −2.9	0.011

## 4 DISCUSSION

Given the rising prevalence of AR, surveillance of test outputs to monitor the effectiveness of current anthelmintic treatments and the prevalence of parasitic infections has become increasingly important.^5^ A review of current diagnostic test and surveillance methods and available surveillance data to understand trends is therefore timely and warranted.

### 4.1 FWECT methods and thresholds used by UK equine diagnostic laboratories

The gold standard test for detecting nematode species in horses and quantifying burdens involves post‐mortem examination, with this being an improbable test choice as a result of its terminal status and cost and logistical limitations.^4^, ^20^, ^21^ Faecal analysis is therefore more appropriate, accessible and widely used. It should be noted that FWECTs have limitations as they are weakly correlated with burden and they rely on the presence of eggs in the faeces and so are unable to evaluate the presence of every type of gastrointestinal parasite.^8^, ^10^, ^11^ Therefore, FWECT results will not reflect the presence of larvae, nor of parasites which do not lay eggs in the equine gastrointestinal tract, such as *Gastrophilus* botflies or encysted cyathostomins.^22^ Further limitations on the interpretation of FWECT include influences such as the immune and nutritional status of the host on the number of eggs shed per adult worm. These influences would not be expected to account for fluctuations in FWECT between years at the UK horse population level. Novel molecular methods conducted on parasites in faecal samples, such as PCR, are available, but are currently limited to a few nematode species.^23^, ^24^ Additionally, next‐generation sequencing is being more widely researched for the purposes of investigating gastrointestinal nematodes in livestock and is an evolving field.^25^ Although testing methods varied among the laboratories contributing to the EQDSR, FWECT using the McMaster method or its modified versions were principally used, as indicated by 92% of 13 survey responding laboratories. Despite being the main test choice, there remains significant variability in how the test is conducted and no formal standardisation of the test method currently exists to optimise test outputs, despite guidelines existing for using FWECRTs.^26^ FWECTs are affected by many variables, some of which may be controllable through standardisation in testing methodology. One such variable includes the sampling method, which is often conducted by horse owners. The survey of laboratories showed variability in the volume of faeces requested by different laboratories, ranging from 2 g to golf ball‐sized amounts, with only 4 of 13 laboratories specifying the need to take samples from various areas across a dropping, despite the varied distribution of eggs in faeces and the need for best practice in sample acquisition.^27^ While information on the multiplication factor used in a McMaster test to estimate the epg showed wide variation across laboratories (median = ×50, range = ×10 to ×100, *n* = 11 laboratories), the laboratory survey did not request any other specific details about FWECT methods, such as sample storage before receipt by the laboratory and the required freshness of a sample, the volume of faeces and flotation solutions used in the methods, all also being variables that may affect test outputs.^28^ A recent systematic review emphasised the lack of standardisation in validating and comparing equine faecal egg count techniques and identified inconsistencies in methods, performance parameters and result interpretation in studies assessing different techniques.^25^ This is highlighted here by two laboratories (A and F) that cited a multiplication factor of 100 epg but specified a lower value of 50 epg for determining a positive test result. Most research studies use the McMaster technique for comparing FWECT performance but generally reported higher accuracy, precision and sensitivity for other alternative techniques. Additionally, sugar‐based solutions were found to be more effective in egg recovery across various methods. Future work is required to further understand the extent of the variability in FWECT methods and encourage standardisation to ensure optimisation of FWECT results.

The lack of consensus on the appropriate epg threshold for reporting positive results to the EQDSR and for determining anthelmintic treatment application highlights a critical gap in standardisation for surveillance and clinical purposes. For surveillance purposes, the most commonly reported threshold value for designating a FWECT as positive for strongyles to the EQDSR was ≥200 epg, being applied by 6 of 18 (33%) responding laboratories. When these values are compared with those levels for which a laboratory would advise treatment of a horse with an anthelmintic, four laboratories submitted surveillance data using the same threshold that they would advise treatment at. For the other five laboratories for which both values were provided, the difference between reporting for surveillance and treatment varied from +100 to +450 epg above the positive threshold epg value. The epg treatment threshold being higher than those used for surveillance reporting are positive in terms of avoiding AR by encouraging selective‐treatment strategies that leave parasites in refugia by using FWECT to identify and treat high‐shedding animals only.^12^ It would be prudent to establish a system in which laboratories share absolute counts of FWECTs data for surveillance purposes and the percentage of these that were recommended for treatment, or ideally that actually received an anthelmintic and the class given. However, inherent issues exist for this given the previously discussed challenges of obtaining such data and that treatment choices may commonly be made by referring veterinary surgeons with no feedback loop to sharing these clinical decisions with the testing laboratory or wider surveillance initiative.

The current lack of standardisation in the FWECT methods and of the thresholds used to interpret results across laboratories makes the output data less reliable and thus prevents valid evaluation of the efficacy of current strategies to combat AR. Guidelines exist to advise on epg thresholds to indicate whether to administer an anthelmintic but it is important that a single FWECT result should not be interpreted in isolation when making treatment decisions and factors including the premises population, management practices and animals' risk profiles should be evaluated too.^29^ This study highlights the current lack of standardisation across the industry in treatment approaches, with vastly different reported epg thresholds used by different laboratories to indicate which animals require treatment. This finding is concerning and UK‐wide industry discussion is ongoing to determine best practice to optimise anthelmintic usage and guidelines^6^, ^30^ (BEVA). Crucially, a detailed analysis of the appropriate thresholds triggering anthelmintic administration and how these thresholds may depend on factors such as the age of the host sampled and estimations of the level of AR within the yard‐level worm population, is urgently needed. Increasing PTP could in turn lead to an increase in the treatment frequency, especially if anthelmintics are administered at inappropriate epg thresholds. There remains a real risk that AR, leading to higher epgs on FWECTs, will ‘feed on itself’ and inadvertently increase the rate of establishment of further AR. Therefore, the optimal threshold for anthelmintic administration may in turn have to be guided by the faecal worm egg count reduction test (FWECRT) carried out for the anthelmintic intended to be used, alongside a comprehensive evaluation of management practices and individual risk profiles. Although not explicitly asked in the questionnaire in this study, five laboratories stated that treatment decisions should be made in consultation with a veterinary surgeon.

Currently, there are clear practical limitations to strongyle surveillance. It is well known that extracting data from laboratory management systems is time‐consuming, which may hinder changing processes in reporting for surveillance purposes. Alongside this, the clinical purpose of sample submission to laboratories was not known and samples may have been submitted to identify animals requiring anthelmintic treatment or for the purposes of evaluation of anthelmintic treatment efficacy by FWECRT. The EQDSR now requests that FWECRT data be shared separately to single FWECT results. No information regarding how laboratories conduct FWECRT were obtained in this study but guidelines exist to encourage standardisation of methods and more effective monitoring of AR.^26^, ^31^

Limited data were collected on ascarids within the questionnaire and most laboratories report and advise treatment for ascarid egg presence in a FWECT at the same positive threshold that they use for strongyles. Currently, the correlation between epg and worm burdens has not been demonstrated for ascarids.^4^, ^11^

### 4.2 FWECT laboratory surveillance data submitted to EQDSR

Overall, there was clear evidence to support the logical assumption that adopting lower FWECT‐thresholds for surveillance reporting resulted in higher PTP (positivity rates), with the highest PTP levels evident among the baseline threshold category (>0 to <100 epg) and a clear trend in regression analysis for reducing PTP with application of increasing threshold levels. Interestingly, the numerically largest NA group for which the threshold was not able to be determined, was also associated with significantly lower positivity rates relative to baseline. This would be consistent with a proportion at least of these 19 laboratories applying higher FWECT‐threshold levels, although it was not possible to investigate this consideration further.

When assessing if a consistent within‐year seasonal pattern existed in this surveillance dataset, the quarter of the year showed no evidence of statistically significant differences in PTP in spring (April–June), summer (July–September) or autumn (October–December) relative to the baseline winter (January–March) category. Obtaining individual FWECT results would enhance the evaluation of seasonality, as quarterly aggregated data significantly reduces temporal resolution. Previous studies globally have found a seasonal trend in FWECTs, with the highest strongyle egg shedding being in the autumn and lower FWECs being observed in spring and winter.^20^, ^25^, ^32^, ^33^, ^34^ Climatic factors like temperature and rainfall influence larval dynamics, impacting faecal egg counts and parasite transmission.^35^, ^36^ The UK Sustainable Control of Parasites in Sheep (SCOPS) initiative identifies high‐risk weather/climate periods and facilitates the implementation of targeted treatment strategies for effectively managing gastrointestinal nematodes.^37^ Consideration on how this sort of system may be adapted for the equine population to establish surveillance to monitor weather patterns and target treatment during higher risk periods is essential in developing a dynamic approach to combat burdens of larval exposure, while mitigating growing AR concerns.

Overall, controlling for between‐laboratory variation and FWECT thresholds, there remained evidence for a significant gradient in increasing PTP over the study period relative to the baseline years of 2007–2009. Determining year‐on‐year temporal trends through prevalence studies presents inherent challenges. Available studies predominantly employ cross‐sectional and longitudinal designs, which often suffer from biases in case selection and small sample sizes.^34^ Collated diagnostic test data, such as EQDSR surveillance data, provide a more accessible but still imperfect dataset. However, biases such as sampling being conducted by conscientious keepers and a higher frequency of samples from clinical cases may affect reliability, in addition to the challenges previously discussed with diagnostic test method variabilities affecting results. Despite the variation in testing choice, the retrospective EQDSR surveillance data does not specify which samples were run using each FWECT method and therefore this must be taken into consideration when interpreting results as this variable was unable to be controlled for during analysis.

Regardless, the consistent increase in the proportion of positive FWECT results over the 17‐year period of EQDSR data suggests a growing number of UK horses with patent infections, where parasitic stages have matured sufficiently to produce detectable eggs. Several factors may explain this trend. Positive‐thresholds used by laboratories for reporting surveillance data varying over time could be a factor, but this has been controlled for in this analysis and the temporal PTP trend remains. AR likely plays a significant role, leading to suboptimal parasite control and greater pasture contamination, thus increasing exposure to infective larvae. Climatic changes, such as warmer and wetter conditions, may also contribute by enhancing larvae survival and speeding up development, further contaminating pastures. Moreover, current selective treatment practices, which aim to slow AR development by treating only the horses shedding the most eggs, may inadvertently lead to higher egg shedding from untreated animals, compounding pasture contamination. These interconnected factors—climate change, AR and selective treatment—create potential enhancing feedback loops. Climate change may further influence the development of AR through the reduction of refugia (e.g., the proportion of worm stages not exposed to the anthelmintic at the time of administration), for example as the result of larval die‐off at pasture during droughts.^38^ The rising proportion of positive samples, regardless of the underlying causes, presents a significant risk for the ongoing development of AR, particularly if it leads to more animals crossing the designated treatment thresholds driving treatment decisions, and highlights the need for continued surveillance and refined management strategies.

This study has several limitations that may impact the interpretation of the results. First, there could have been multiple samples from the same animal in the dataset, taken at different time points. With no ability to track these repeated samples, data may not fully capture the true burden of infection or the effectiveness of treatment over time. Moreover, the absence of background data for samples, such as equine age, reason for testing, case location and prior anthelmintic use history are limiting factors. Age is particularly important, as youngstock are known to have higher mean strongyle egg counts compared with adult horses and do not fit the typical 80:20 rule, where a minority of the population sheds the majority of the eggs.^39^, ^40^, ^41^ Without this demographic information, it is challenging to fully assess the factors contributing to positive FWECT results, particularly in distinguishing age‐related shedding patterns. This lack of context limits the ability to make more targeted recommendations or draw conclusions about the broader population dynamics of parasitic infection and resistance development. Additionally, the spatial distribution of the laboratories contributing surveillance data demonstrates clustering in Southern and Central England. This uneven distribution could skew results, especially if these laboratories serve areas with denser equine populations. Although postal samples are received from across the United Kingdom, mitigating some geographic bias, the absence of detailed information on sample origins limits understanding of the dataset's spatial representativeness. Finally, the fact that test results are only provided as positive or negative, without quantifiable data on epg, further limits the sensitivity of analyses. This lack of precision hinders the ability to conduct more detailed studies on infection intensity and its implications for resistance management.

The survey data collected from laboratories conducting FWECT had a good response rate but the absence of comprehensive survey data for the whole 17‐year surveillance period to assist in data interpretation is likely to affect the completeness and representativeness of our findings. Challenges arose as laboratories previously supplying data may have either stopped conducting FWECT or no longer exist; for example, the Animal Health Trust, which provided data from the inception of the EQDSR, permanently closed in July 2020. In addition, one laboratory supplied surveillance data of only the number tested and not the number positive, due to challenges in obtaining the data, demonstrating the potential issues that could be faced if amendments are made to refine data quality and usability. Additional amendments to the way data are collected for the purposes of surveillance warrant further discussion to overcome these limitations. Considering using proposed risk categories and incorporating case and population data alongside FWECT results would be the optimal approach but may suffer from logistical challenges in obtaining such data. Going forward, an epg threshold for reporting a positive result to the EQDSR should be agreed upon and ideally this should be comparable to the epg level that would be used to advise treatment in most scenarios, dependent on a standardised method for identifying a case's risk category.^29^ The hope is that this would unite efforts on reducing AR and create more accurate data for the EQDSR and for surveillance, which would guide future strategies.

## 5 CONCLUSION

Despite there being significant variation in diagnostic laboratory testing methods for FWECT which requires standardisation, the EQDSR surveillance data reveal an increase in the proportion of positive FWECT results among UK horses from 2007 to 2023. While improved testing practices may contribute to more positive identifications, such findings suggest a potential crisis in equine health, as increased egg shedding could signal rising parasite burdens and heighten concerns over AR. These data highlight an urgent need for comprehensive investigations into the underlying factors driving these trends. Immediate action is essential to develop effective strategies for managing parasite burdens and addressing the threat of AR in the equine population.

## FUNDING INFORMATION

The UK's Department for Environment, Food and Rural Affairs (Defra) support the production of the Equine Quarterly Disease Surveillance Report. FW and RN are supported through a combined contribution to Equine Infectious Disease Surveillance (EIDS) from the Horserace Betting Levy Board (HBLB), the Racehorse Owners' Association (ROA) and the Thoroughbred Breeders' Association (TBA).

## CONFLICT OF INTEREST STATEMENT

The authors have declared no conflicting interests.

## AUTHOR CONTRIBUTIONS

**Fleur Whitlock:** Investigation; methodology; validation; visualization; writing – review and editing; software; formal analysis; data curation; writing – original draft; project administration. **Jan van Dijk:** Writing – review and editing; validation; conceptualization. **Jane E. Hodgkinson:** Validation; writing – review and editing. **John Duncan Grewar:** Writing – review and editing; visualization; validation; software; formal analysis; data curation. **J. Richard Newton:** Conceptualization; investigation; funding acquisition; writing – original draft; methodology; validation; project administration; formal analysis; visualization; writing – review and editing; supervision; resources.

## DATA INTEGRITY STATEMENT

Fleur Whitlock and Richard Newton had full access to all the data in the study and take responsibility for the integrity of the data and the accuracy of data analysis.

## ETHICAL ANIMAL RESEARCH

This work was approved by the University of Cambridge's Department of Veterinary Medicine Ethics and Welfare Committee (CR829).

## INFORMED CONSENT

Explicit owner consent for animals' inclusion in the study was not stated.

## PEER REVIEW

The peer review history for this article is available at https://www.webofscience.com/api/gateway/wos/peer-review/10.1111/evj.14478.

## ANTIMICROBIAL STEWARDSHIP POLICY

Not applicable.

## Supporting information


**Data S1.** Questionnaire S1: Laboratory faecal worm egg count test (FWECT) methods questionnaire sent to 17 laboratories contributing FWECT data to Equine Quarterly Disease Surveillance Report in Q4 2023.

## Data Availability

The data that support the findings of this study are openly available at https://github.com/jdatarsa/data_fwect.

## References

[1] Cai E , Wu R , Wu Y , Gao Y , Zhu Y , Li J . A systematic review and meta‐analysis on the current status of anthelmintic resistance in equine nematodes: a global perspective. Mol Biochem Parasitol. 2024;257:111600. 10.1016/j.molbiopara.2023.111600 38030084

[2] Peregrine AS , Molento MB , Kaplan RM , Nielsen MK . Anthelmintic resistance in important parasites of horses: does it really matter? Vet Parasitol. 2014;201(1–2):1–8. 10.1016/j.vetpar.2014.01.004 24485565

[3] Nielsen MK . Anthelmintic resistance in equine nematodes: current status and emerging trends. Int J Parasitol Drugs Drug Resist. 2022;20:76–88. 10.1016/j.ijpddr.2022.10.005 36342004 PMC9630620

[4] Reinemeyer CR . Diagnosis and control of anthelmintic‐resistant *Parascaris equorum* . Parasit Vectors. 2009;2:2. 10.1186/1756-3305-2-S2-S8 PMC2751844 19778469

[5] Kaplan RM , Vidyashankar AN . An inconvenient truth: global worming and anthelmintic resistance. Vet Parasitol. 2012;186(1–2):70–78. 10.1016/j.vetpar.2011.11.048 22154968

[6] CANTER . Controlling ANTiparasitic Resistance in Equines Responsibly. https://canterforhorses.org.uk/

[7] EIDS . Equine Quarterly Disease Surveillance Report, Produced by Equine Infectious Disease Surveillance (EIDS). https://equinesurveillance.org/

[8] Becher AM , Mahling M , Nielsen MK , Pfister K . Selective anthelmintic therapy of horses in the Federal states of Bavaria (Germany) and Salzburg (Austria): an investigation into strongyle egg shedding consistency. Vet Parasitol. 2010;171(1–2):116–122. 10.1016/j.vetpar.2010.03.001 20356680

[9] Nielsen MK , Haaning N , Olsen SN . Strongyle egg shedding consistency in horses on farms using selective therapy in Denmark. Vet Parasitol. 2006;135(3–4):333–335. 10.1016/j.vetpar.2005.09.010 16226379

[10] Duncan JL . Field studies on the epidemiology of mixed strongyle infection in the horse. Vet Rec. 1974;94(15):337–345. 10.1136/vr.94.15.337 4836097

[11] Nielsen MK , Baptiste KE , Tolliver SC , Collins SS , Lyons ET . Analysis of multiyear studies in horses in Kentucky to ascertain whether counts of eggs and larvae per gram of feces are reliable indicators of numbers of strongyles and ascarids present. Vet Parasitol. 2010;174(1–2):77–84. 10.1016/j.vetpar.2010.08.007 20850927

[12] Van Wyk JA . Refugia – overlooked as perhaps the most potent factor concerning the development of anthelmintic resistance. Onderstepoort J Vet Res. 2001;68(1):55–67. 11403431

[13] Gomez HH , Georgi JR . Equine helminth infections: control by selective chemotherapy. Equine Vet J. 1991;23(3):198–200. 10.1111/j.2042-3306.1991.tb02754.x 1884701

[14] Duncan JL , Love S . Preliminary observations on an alternative strategy for the control of horse strongyles. Equine Vet J. 1991;23(3):226–228. 10.1111/j.2042-3306.1991.tb02762.x 1909236

[15] Lester HE , Matthews JB . Faecal worm egg count analysis for targeting anthelmintic treatment in horses: points to consider. Equine Vet J. 2014;46(2):139–145. 10.1111/evj.12199 24131301

[16] Gordon HM , Whitlock AV . A new technique for counting nematode eggs in sheep feces. J Council Sci Ind Res. 1939;12(1):50–52.

[17] Whitlock HV . Some modifications of the McMaster helminth egg‐counting technique and apparatus. J Council Sci Ind Res. 1948;21:177–180.

[18] Zoetis Ovactec Plus . https://www2.zoetis.nl/diersoort/paarden/products/ovatec-plus

[19] Ovacyte . https://www.telenostic.com/about-ovacyte/

[20] Mfitilodze MW , Hutchinson GW . The site distribution of adult strongyle parasites in the large intestines of horses in tropical Australia. Int J Parasitol. 1985;15(3):313–319. 10.1016/0020-7519(85)90069-4 4030206

[21] Sallé G , Guillot J , Tapprest J , Foucher N , Sevin C , Laugier C . Compilation of 29 years of postmortem examinations identifies major shifts in equine parasite prevalence from 2000 onwards. Int J Parasitol. 2020;50(2):125–132. 10.1016/j.ijpara.2019.11.004 31981673

[22] Dowdall SMJ , Matthews JB , Mair T , Murphy D , Love S , Proudman CJ . Antigen‐specific IgG(T) responses in natural and experimental cyathostominae infection in horses. Vet Parasitol. 2002;106(3):225–242. 10.1016/S0304-4017(02)00085-7 12062511

[23] Roeber F , Morrison A , Casaert S , Smith L , Claerebout E , Skuce P . Multiplexed‐tandem PCR for the specific diagnosis of gastrointestinal nematode infections in sheep: an European validation study. Parasit Vectors. 2017;10(1):226. 10.1186/s13071-017-2165-x 28482924 PMC5422907

[24] Reslova N , Skorpikova L , Kyrianova IA , Vadlejch J , Höglund J , Skuce P , et al. The identification and semi‐quantitative assessment of gastrointestinal nematodes in faecal samples using multiplex real‐time PCR assays. Parasit Vectors. 2021;14(1):391. 10.1186/s13071-021-04882-4 34372893 PMC8351436

[25] Abbas G , Ghafar A , Beasley A , Stevenson MA , Bauquier J , Koehler AV , et al. Understanding temporal and spatial distribution of intestinal nematodes of horses using faecal egg counts and DNA metabarcoding. Vet Parasitol. 2024;325:110094. 10.1016/j.vetpar.2023.110094 38091893

[26] Kaplan RM , Denwood MJ , Nielsen MK , Thamsborg SM , Torgerson PR , Gilleard JS , et al. World Association for the Advancement of Veterinary Parasitology (W.A.A.V.P.) guideline for diagnosing anthelmintic resistance using the faecal egg count reduction test in ruminants, horses and swine. Vet Parasitol. 2023;318:109936. 10.1016/j.vetpar.2023.109936 37121092

[27] Lester H , Bartley D , Morgan E , Hodgkinson J , Matthews J . The spatial distribution of strongyle eggs in horse faeces. J Equine Vet Sci. 2012;32(10):S33–S34. 10.1016/j.jevs.2012.08.077

[28] Nielsen MK , Vidyashankar AN , Andersen UV , DeLisi K , Pilegaard K , Kaplan RM . Effects of fecal collection and storage factors on strongylid egg counts in horses. Vet Parasitol. 2010;167(1):55–61. 10.1016/j.vetpar.2009.09.043 19850412

[29] Rendle D , Austin C , Bowen M , Cameron I , Furtado T , Hodgkinson J , et al. Equine de‐worming: a consensus on current best practice. UK‐Vet Equine. 2019;10.12968/ukve.2019.3.s.3:3–14.

[30] Rendle D , Hughes K , Bowen M , Bull K , Cameron I , Furtado T , et al. BEVA primary care clinical guidelines: Equine parasite control. Equine Vet J. 2024;56(3):392–423. 10.1111/evj.14036 38169127

[31] Geurden T , Olson ME , O'Handley RM , Schetters T , Bowman D , Vercruysse J . World Association for the Advancement of Veterinary Parasitology (WAAVP): guideline for the evaluation of drug efficacy against non‐coccidial gastrointestinal protozoa in livestock and companion animals. Vet Parasitol. 2014;204(3–4):81–86. 10.1016/j.vetpar.2014.02.050 25285343

[32] Beasley AM , Kotze AC , Barnes TS , Coleman GT . Equine helminth prevalence and management practices on Australian properties as shown by coprological survey and written questionnaire. Anim Prod Sci. 2020;60(18):2131–2144. 10.1071/AN18378

[33] Sargison N , Chambers A , Chaudhry U , Costa Júnior L , Doyle SR , Ehimiyein A , et al. Faecal egg counts and nemabiome metabarcoding highlight the genomic complexity of equine cyathostomin communities and provide insight into their dynamics in a Scottish native pony herd. Int J Parasitol. 2022;52(12):763–774. 10.1016/j.ijpara.2022.08.002 36208676

[34] Boelow H , Krücken J , von Samson‐Himmelstjerna G . Epidemiological study on factors influencing the occurrence of helminth eggs in horses in Germany based on sent‐in diagnostic samples. Parasitol Res. 2023;122(3):749–767. 10.1007/s00436-022-07765-4 36627515 PMC9988789

[35] Baudena MA , Chapman MR , French DD , Klei TR . Seasonal development and survival of equine cyathostome larvae on pasture in south Louisiana. Vet Parasitol. 2000;88(1–2):51–60. 10.1016/S0304-4017(99)00198-3 10681022

[36] Ramsey YH , Christley RM , Matthews JB , Hodgkinson JE , McGoldrick J , Love S . Seasonal development of Cyathostominae larvae on pasture in a northern temperate region of the United Kingdom. Vet Parasitol. 2004;119(4):307–318. 10.1016/j.vetpar.2003.11.014 15154595

[37] Taylor MA . SCOPS and COWS—worming it out of UK farmers. Vet Parasitol. 2012;186(1–2):65–69. 10.1016/j.vetpar.2011.11.047 22222010

[38] Knapp‐Lawitzke F , von Samson‐Himmelstjerna G , Demeler J . Elevated temperatures and long drought periods have a negative impact on survival and fitness of strongylid third stage larvae. Int J Parasitol. 2016;46(4):229–237. 10.1016/j.ijpara.2015.10.006 26828893

[39] Relf VE , Morgan ER , Hodgkinson JE , Matthews JB . Helminth egg excretion with regard to age, gender and management practices on UK Thoroughbred studs. Parasitology. 2013;140(5):641–652. 10.1017/S0031182012001941 23351718

[40] Lester HE , Morgan ER , Hodgkinson JE , Matthews JB . Analysis of strongyle egg shedding consistency in horses and factors that affect it. J Equine Vet Sci. 2018;60:113–119.e1. 10.1016/j.jevs.2017.04.006

[41] Wood EL , Matthews JB , Stephenson S , Slote M , Nussey DH . Variation in fecal egg counts in horses managed for conservation purposes: individual egg shedding consistency, age effects and seasonal variation. Parasitology. 2013;140(1):115–128. 10.1017/S003118201200128X 22894917

