# Supplementary material for: Reasons to be fearful? Rising proportions of positive faecal worm egg counts among UK horses (2007–2023)
Source: Equine Vet J. 2025 Jan 22;57(6):1572–83. doi: 10.1111/evj.14478 (PMC12508284; doi:10.1111/evj.14478)
Supplement: Supplementary file 1 — Data S1. Questionnaire S1: Laboratory faecal worm egg count test (FWECT) methods questionnaire sent to 17 laboratories contributing FWECT data to Equine Quarterly Disease Surveillance Report in Q4 2023. [file EVJ-57-1572-s001.pdf]

Equine Veterinary Journal BEVA

Questionnaire S1: Laboratory faecal worm egg count test methods questionnaire sent to 17 laboratories contributing faecal worm egg count test data to Equine Quarterly Disease Surveillance report in Q4 2023.

Laboratory:		
Name of responder:		
Question	Strongyles	Ascarids
What methods of FWEC testing do you conduct in your lab?		
What volume of faeces do you request owners to collect for testing?		
What multiplication factor do you use to estimate eggs per gram?		
When reporting results, what eggs per gram value would you specify as positive for the purpose of supplying data to the Equine Quarterly Report?		
At what eggs per gram level would you be advising treating with an anthelmintic?		
What comments to you utilise when reporting results to your clients?		
